# Clinical Performance Evaluation of the NeuMoDx Flu A-B/RSV/SARS-CoV-2 Vantage Assay

**DOI:** 10.3390/diagnostics12123201

**Published:** 2022-12-16

**Authors:** Georgios Meletis, Areti Tychala, Ioanna Gkeka, Athanasia Gkotzia, Aikaterini Triantafyllou, Styliani Pappa, Maria Exindari, Georgia Gioula, Anna Papa, Lemonia Skoura

**Affiliations:** 1 Department of Microbiology, AHEPA University Hospital, Medical School, Aristotle University οf Thessaloniki, 54636 Thessaloniki, Greece; 2 Laboratory of Microbiology, Medical School, Aristotle University of Thessaloniki, 54636 Thessaloniki, Greece

**Keywords:** SARS-CoV-2, RSV, Influenza, syndromic testing

## Abstract

SARS-CoV-2 infections may present with various symptoms that are similar to those of other respiratory diseases. For this reason, the need for simultaneous detection of at least RSV and influenza viruses together with SARS-CoV-2 was evident from the early stages of the pandemic. In the present study, we evaluated the clinical performance of the NeuMoDx™ Flu A-B/RSV/SARS-CoV-2 Vantage Assay against the conventional low-plex PCR utilized to detect influenza A-B, RSV, and SARS-CoV-2. There were 115 known positive clinical samples and 35 negative controls obtained from asymptomatic health-care workers included in the study; 25 samples were positive for influenza viruses, 46 for RSV, and 44 for SARS-CoV-2. The sensitivity, specificity, positive predictive value, and negative predictive value of the evaluated method for influenza and SARS-CoV-2 were 100%. The Spearman correlation coefficient was 0.586 (*p* < 0.05) for influenza and 0.893 (*p* < 0.05) for SARS-CoV-2. The sensitivity of the aforementioned assay for RSV was 93.47%; the specificity and the positive predictive value were 100%, and the negative predictive value was 92.10%, while the Spearman correlation coefficient was not applicable for the RSV. Overall, the assay under evaluation was shown to be a reliable alternative for the simultaneous detection of influenza viruses, RSV and SARS-CoV-2.

## 1. Introduction

Severe acute respiratory syndrome coronavirus 2 (SARS-CoV-2) has spread worldwide, causing the coronavirus disease (COVID-19) pandemic with millions of casualties and unprecedented socio-economic effects [1]. COVID-19 presents with a range of symptoms, and the disease ranges from asymptomatic or a flu-like illness, to acute respiratory distress syndrome [2]. Therefore, it cannot be easily distinguished from other respiratory diseases like influenza and respiratory syncytial virus (RSV) infection without laboratory testing. Thus, excessive testing for SARS-CoV-2 has been one of the most valuable tools to control the pandemic and guide public health measures [3].

During the cold season of 2020–2021, influenza viruses and RSV did not actually occur within their usual seasonality [4]. In the northern hemisphere, RSV cases started increasing paradoxically from spring to summer 2021, depending on the region [4,5,6], and then an unexpected early sudden upsurge was observed in autumn 2022 [7], while influenza, following a year of absence, appeared again in winter 2022, though in lowerprevalencethaninpro-COVID-19 years [8]. This can be attributed to the use of masks, social distancing, excessive disinfection, and strict lockdowns. Another reason could be the intra-host viral interference that can suppress a virus in favor of the dominant one, as well as a result in shifts of epidemic curves [9].

In spite of the clinical similarities, these three respiratory infections require different management when it comes to infection, control measures, and therapeutics. More specifically, in cases of SARS-CoV-2, influenza A, B, and RSV infections, airborne, droplet and contact precautions need to be applied, respectively [10,11]. Additionally, for influenza patients, especially those admitted to hospital and those belonging to special populations, such as the immunocompromised, a prompt antiviral treatment is required [12].

Another aspect that seems to be worrisome is the appearance of SARS-CoV-2 co-infections with influenza viruses and RSV. Several reports worldwide since the beginning of the pandemic indicate this fact, although the full extent of co-infections cannot be estimated due to very limited testing data. These co-infections, especially those with influenza viruses, may result insubstantial morbidity and mortality increase [13,14,15].

According to the ECDC and WHO, it is crucial, especially during influenza season, all patients with acute respiratory symptoms in hospitals and in other healthcare settings, and all specimens from sentinel primary care surveillance, to be tested for both SARS-CoV-2 and seasonal influenza, while there is a need to monitor in parallel the incidence and trends over time and to apply response measures in a consistent manner [16,17].

Thus, the ongoing need for COVID-19 diagnostics, notably in symptomatic patients, and the clinical similarities to flu and RSV infection raises the need for rapid, yet accurate, differentiation among these infections in order to address infection control, treatment and surveillance issues accordingly.

In this context, different commercial multiplex polymerase chain reaction (PCR) assays combined testing for influenza, respiratory syncytial virus (RSV) and SARS-CoV-2 have received the emergency use authorization (EUA) by the FDA. Among them, the NeuMoDx™ Flu A-B/RSV/SARS-CoV-2 Vantage Assay is a new respiratory test launched by Qiagen, which takes advantage of the NeuMoDx 96 and NeuMoDx 288 high throughput fully automated one-step systems’ that provides results in less than three hours [18].

The aim of our study was to evaluate the clinical performance of the NeuMoDx™ Flu A-B/RSV/SARS-CoV-2 Vantage Assay for the simultaneous detection of the three viruses, influenza A/B, SARS-VoV-2, and RSVin respiratorysamples.

## 2. Materials and Methods

### 2.1. Study Sample and Setting

In total, we tested 150 respiratory samples; 115 known positive remnant samples and 35 negative controls collected from asymptomatic health-care workers, in the context of screening testing for SARS-CoV-2 from 1 to 15 May 2022, after the end of 2021–2022 flu season. Among the positive samples, 25 were positive for influenza viruses; 46 were positive for RSV and 44 were positive for SARS-CoV-2. One sample (ID 83547) was positive for both RSV and SARS-CoV-2. All were nasopharyngeal swab (NP) samples and after processing were stored at −80 °C until further examination. The multiplex PCR and PCR for SARS-CoV-2 detection took place at the Molecular diagnostics Laboratory of the AHEPA University Hospital in Thessaloniki, and the respective PCRs for influenza and RSV were performed at the Laboratory of Microbiology of Medical School of the Aristotle University of Thessaloniki in Greece.

### 2.2. NeuMoDx Flu A-B/RSV/SARS-CoV-2 Vantage Assay

The NeuMoDx™ Flu A-B/RSV/SARS-CoV-2 Vantage Assay (QIAGEN, USA) was performed on the NeuMoDx™ 96 Molecular System using the pretreated workflow. It is a multiplex, rapid and automated qualitative in vitro real-time RT-PCR diagnostic test intended for simultaneous direct detection and differentiation of influenza A virus, influenza B virus, RSV and SARS-CoV-2 from NP swab samples in transport medium. The assay combines automated RNA extraction and amplification/detection of viruses by real-time RT-PCR. The NeuMoDx System uses a combination of heat, lytic enzyme, and extraction reagents to automatically perform lysis, RNA extraction, and removal of inhibitors. The released nucleic acids are captured by paramagnetic particles and the NeuMoDx System enables simultaneous amplification and detection of all targets and sample process control RNA sequences. An important feature of the assay is that samples can be processed individually without the use of sample plates. The limit of detection (LoD) is 0.5 TCID_50_/mL for influenza A and RSV, 0.25 TCID_50_/mL for influenza B, and 150 copies/mL for SARS-CoV-2 [19].

More precisely, the NeuMoDx Molecular System automatically performs all the steps required to extract the target nucleic acid, prepare the isolated RNA for real-time reverse transcriptase polymerase chain reaction (RT-PCR) and, if present, amplify and detect the products of amplification. The NeuMoDx Flu A-B/RSV/SARS-CoV-2 Vantage Assay targets the conserved region of SARS-CoV-2 Nsp2 gene and regions in the M genes of influenza A, influenza B and respiratory syncytial virus A or B genomes. The NeuMoDx Flu A-B/RSV/SARS-CoV-2 Vantage Assay includes an RNA Sample Process Control (SPC2) to help monitor for the presence of potentially inhibitory substances and NeuMoDx System or reagent failures that may be encountered during the extraction and amplification process. The amplification process includes 50 PCR cycles.

### 2.3. Influenza Type A and B RT-PCR

RNA was extracted using the Qiagen Viral RNA mini kit (Qiagen, Hilden, Germany) according to the manufacturer’s instructions. The detection of influenza type A and B viruses, the subtyping of influenza A (H3 or H1pdmo09) and the B virus lineage-genotyping were performed by real-time one step RT-PCRs with specific primers and probes provided to the National Influenza Centre of N. Greece by the International Reagent Resource (https://www.internationalreagentsource.org (accessed on 3 November 2022)) using the CDC influenza real-time RT-PCR protocol, following WHO recommendations (CDC Real-time RT-PCR protocol for detection and characterization of swine influenza, version 2009) (https://cdn.who.int/media/docs/default-source/influenza/molecular-detention-of-influenza-viruses/protocols_influenza_virus_detection_feb_2021.pdf?sfvrsn=df7d268a_5 (accessed on 3 November 2022)).

### 2.4. RSV RT-Nested PCR

RNA was extracted using the Qiagen Viral RNA mini kit (Qiagen, Hilden, Germany) according to the manufacturer’s instructions. The detection of RSV and the simultaneously identification of the virus subtypes (A and B) were performed by using an RT-nested PCR, which amplifies a highly conserved region of the RSV fusion protein gene; the identification of the subtype was performed on the basis of the size of the PCR product (363 bp and 611 bp for RSV-A and RSV-B, respectively) [20]. For further genotyping, two one-step RT-PCRs were applied, which amplify a fragment of the second hypervariable region of the G protein gene [21]; PCR products were Sanger sequenced and the sequences were analyzed using the Basic Local Alignment Search Tool (https://blast.ncbi.nlm.nih.gov/ (accessed on 10 December 2021)).

### 2.5. Abbott RealTime SARS-CoV-2 Assay

The nucleic acid extraction and PCR plate setup procedures were performed on the automated m2000sp system, whereas real-time PCR amplification and detection were done on the automated m2000rt system (Abbott, East Touhy Avenue Des Plaines, IL, USA). The target regions of the method are the RNA-dependent RNA polymerase (RdRp) and N genes of SARS-CoV-2 and the LoD is 100 copies/mL [22].

In this method, an RNA sequence that is unrelated to the SARS-CoV-2 target sequence is introduced into each specimen at the beginning of sample preparation. This unrelated RNA sequence is simultaneously amplified by RT-PCR and serves as an internal control (IC) to demonstrate that the process has proceeded correctly for each sample. The IC target sequence is derived from the hydroxypyruvatereductase gene from the pumpkin plant, *Cucurbita pepo*, and is delivered in an Armored RNA^®^ particle that has been diluted in negative human plasma. The Abbott RealTime SARS-CoV-2 assay detects the SARS-CoV-2 virus and IC target sequences through the use of targetspecific fluorescentlabeled oligonucleotide probes. The probes do not generate a signal unless they are specifically bound to the amplified product. The two SARS-CoV-2-specific probes are labeled with the same fluorophore and the IC-specific probe is labeled with a different fluorophore, thus allowing for simultaneous detection of both SARS-CoV-2 and IC amplified products in the same reaction well. This way, a unique Ct value is available for positive samples despite the fact that two SARS-CoV-2 genes are targeted. The assay performs 35 PCR cycles and results with Cts ≥ 20 are considered to refer to low viral load samples.

### 2.6. Statistical Analysis

Correlations between Ct values of the assays were performed using the non-parametric Spearman’s correlation coefficient. A 2-tailed *p* < 0.05 was considered statistically significant. Analyses were performed using the SPSS version 21.

## 3. Results

**Figure 1:**
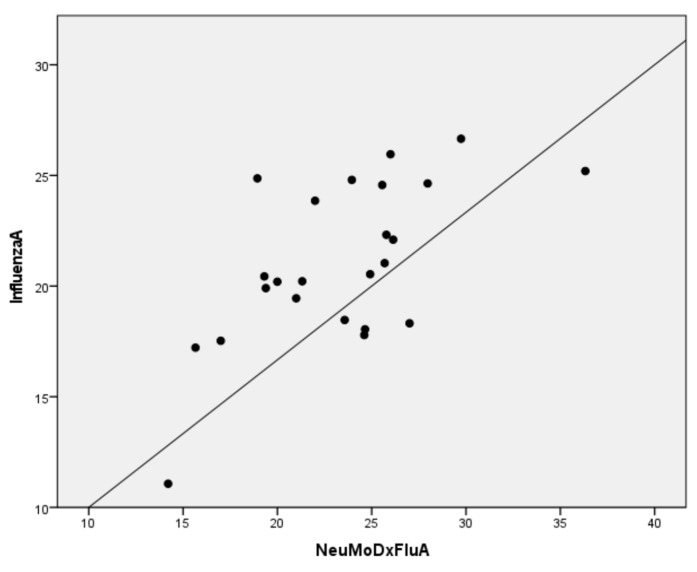
Correlation plots between influenza A RT-PCR and NeuMoDx Flu A results.

All negative controls resulted negative for all four targets of NeuMoDx Flu A-B/RSV/SARS-CoV-2. All influenza positive samples (twenty-four for Flu A and one for Flu B) were positive by the NeuMoDx Flu A-B/RSV/SARS-CoV-2 assay (Table 1). The cycle threshold values (Ct) of positive influenza ART-PCR samples ranged from 11.07 to 26.66 and for the NeuMoDx Flu A they ranged from 14.21 to 36.33.Using as gold standard the CDC influenza real-time RT-PCR kit, the sensitivity, specificity, positive predictive value, and negative predictive value of the method for influenza viruses were 100%. The distributions of the variables were not normal, and the Spearman correlation coefficient was 0.586 (*p* < 0.05) showing moderate positive correlation between Influenza ART-PCR and NeuMoDx Flu A results (Figure 1).

Forty-three out of 46 RSV positive samples were positive by the NeuMoDx Flu A-B/RSV/SARS-CoV-2 assay, including one sample (83547) that resulted positive for both RSV and SARS-CoV-2 (Table 2).This co-infection was previously confirmed by the FilmArray respiratory panel (Biofire Diagnostics) [7].Using as gold standard the RT-nested PCR, the specificity and the sensitivity for RSV of the assay was 100% and 93.47%, respectively, whereas, the positive predictive value was 100% and the negative predictive value was 92.10%. The discordant three RSV samples that resulted negative by the NeuMoDx Flu A-B/RSV/SARS-CoV-2 Assay were also negative in the RSV typing PCR, being positive only in the first RSV diagnostic PCR, suggesting that the viral load was very low.

**Figure 2:**
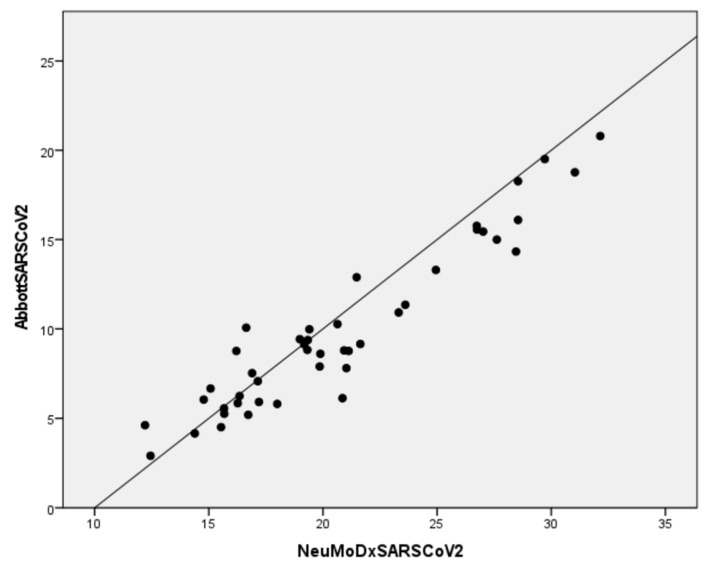
Correlation plots between Abbott RealTime SARS-CoV-2 and NeuMoDx SARS-CoV-2 results.

All forty-four SARS-CoV-2 positive samples were positive by the NeuMoDx Flu A-B/RSV/SARS-CoV-2 assay (Table 3). The Ct values of positive Abbott RealTime SARS-CoV-2 samples ranged from 2.91 to 20.80, and for the NeuMoDx SARS-CoV-2 they ranged from 12.21 to 32.15. Using as gold standard the Abbott RealTime SARS-CoV-2, the sensitivity, specificity, positive predictive value, and negative predictive value of the Assay for SARS-CoV-2 were 100%. The distributions of the variables were not normal, and the Spearman correlation coefficient was 0.893 (*p* < 0.05) indicating strong positive correlation between Abbott RealTime SARS-CoV-2 and NeuMoDxSARS-CoV-2 results (Figure 2).

## 4. Discussion

The COVID-19 pandemic has highlighted the urgency for accurate, reliable, easy to perform, and fast diagnostic tests to upgrade the role of diagnostic laboratories in the management of current healthcare challenges. In this regard, the usefulness of a syndromic approach for the laboratory diagnosis of respiratory tract infections has become evident more than ever [23]. Due to the fact that different viruses cause a broad range of similar symptoms, it is not easy to identify the etiological agent and, thus, many cases remain undiagnosed. A timely and accurate diagnosis, however, may be beneficial for both the patient and the health-care system. Appropriate treatment decisions can reduce the in-hospital stay and, consequently, reduce the risk for nosocomial infections and hospitalization cost. Moreover, the reduction of hospitalizations would be beneficial to contain the ongoing problem of antibiotic resistance among relevant bacterial nosocomial pathogens. Indeed, antibiotic resistance rates have risen significantly during the COVID-19 pandemic because of bacterial co-infections, prolonged hospitalizations and antibiotic overuse [24].

During the pandemic, significant progress has been made in the field of molecular diagnostics and many manufacturers have produced commercial kits for the rapid and simultaneous detection of different respiratory pathogens. Despite being commercially available for some time now, these methodologies have not been yet widely used in laboratory settings due to their cost and because further validation of their performance with real life samples is needed.

It is crucial for clinical microbiology laboratories to safely incorporate new assays in their routine, especially in the lack of reference methods and resources required to proceed to individual assay verifications. In the present study we evaluated the performance of the NeuMoDx Flu A-B/RSV/SARS-CoV-2 Vantage Assay using samples previously tested by routine methods for each pathogen that were used as reference standards. This is the first, to our knowledge, evaluation of the NeuMoDx Flu A-B/RSV/SARS-CoV-2 Vantage Assayperformance against one reference test for each target. With this approach, it was possible to assess the sensitivity, specificity, positive predictive value, and negative predictive value for each virus. The overall results were excellent for influenza and SARS-CoV-2; the results regarding RSV were also very good, as only 3/46 samples with low viral load were not detected. In total, our results are in accordance with a recent study that evaluated the performance of the NeuMoDx Flu A-B/RSV/SARS-CoV-2 Vantage Assay compared to standard of care methods for the detection and differentiation of influenza A and B viruses, RSV, and SARS-CoV-2 with100% agreement [25]. Other commercial multiplex PCR tests targeting influenza A and B viruses, RSV, and SARS-CoV-2 that have been evaluated show similar performance to the NeuMoDx Flu A-B/RSV/SARS-CoV-2 Vantage Assay. For example, the Cepheid Xpert^®^ Xpress SARS-CoV-2/Flu/RSV at a multi center study exhibited 98.2% concordance in clinical samples [26] whereas it has been shown to perform well not only in nasopharyngeal but also in lower respiratory tract specimens [27]. Additionally, Farfour et al. at the Idylla™ SARSCoV2/Flu/RSV evaluation, also showed a good performance of the assay with 82.5% sensitivity, but they as well address the issue of false negative results in low viral loads [28].

During the COVID-19 pandemic, the occurrence patterns of other respiratory pathogens differed from previous years [29]. It is logic to presume that because of the public health measures implemented by the countries to reduce the spread of SARS-CoV-2, the transmission of other respiratory viruses has been reduced, as well [30]. However, influenza A and B viruses and RSV continue to cause seasonal epidemics, and their co-circulation with SARS-CoV-2variants complicates the diagnostic strategies. Moreover, co-infections with SARS-CoV-2 and other respiratory viruses, like that of case ID 83547 cannot be excluded as it is also suggested by Kozinska et al. who found 1.87% of such co-infections among 910 samples were positive for respiratory viruses, from November 2020 to March 2021 [31]. In this case, current data were not made explicit if co-infection drives worse clinical outcomes [32,33,34].

It is expected that as countries decrease the implementation of restricting measures, SARS-CoV-2 will circulate with other respiratory viruses, increasing the probability of co-infections especially during the next winter seasons. Indeed, the actual co-infection rates may already be higher than believed [35,36]. The severity of these co-infections is difficult to predict but recent data suggest cautiousness (including vaccinations and syndromic testing) mostly for influenza/SARS-CoV-2 co-infections [13]. In order to timely detect these cases, some experts in the field supported that all hospitalized COVID-19 patients should be tested for at least influenza at a routine basis [37]. Moreover, in a recent systematic review and meta-analysis it was shown that COVID-19 with other viral co-infections may be associated with adverse clinical outcomes [38].

For the above mentioned reasons a syndromic approach for pathogens causing respiratory symptoms is needed. The implementation of syndromic multiplex panels which include SARS-CoV-2, RSV and influenza viruses is of utmost importance especially for pediatric patients, as they are more likely to be infected by RSV and influenza type B (not excluding type A), while the SARS-CoV-2 symptomatology is milder in them than in adults [39]. The diagnostic challenge is present in several other syndromes, e.g., acute infections of central nervous system, since abroad range of pathogens cause similar symptoms; in all these cases the syndromic approach enables the early and accurate etiological diagnosis for the benefit for the patient and for the public health [40].

A limitation of our study was the use of archived frozen rather than fresh samples which needed to be thawed for reprocessing, whereas there was not enough volume left for additional testing; this could explain the negative result taken in the three RSV-low-positive samples. Moreover, the gene targets used by each platform and the chemistry behind each technology may have biased at some extend the results, especially the Ct value comparisons. Their clinical significance however, remains unequivocal.

## 5. Conclusions

Simultaneous testing for a variety of pathogens inpatients presenting with respiratory tract symptoms is extremely useful for the appropriate treatment of the patient and prompt application of infection control measures. According to the results of the present study, the NeuMoDx Flu A-B/RSV/SARS-CoV-2 Vantage Assay is a reliable alternative for syndromic testing of influenza viruses, RSV, and SARS-CoV-2. Although a plethora of pathogens can cause acute respiratory infections, the Assay covers the testing of the three most often circulating respiratory viruses, and it is expected to be of great help in nosocomial settings.

## Figures and Tables

**Table 1:** Cycle threshold values of influenza A and B RT-PCR positive samples tested on the NeuMoDx Flu A-B/RSV/SARS-CoV-2 Vantage Assay (*n* = 25).

Sample ID	Virus Type	Influenza A and B RT-PCR	NeuMoDx Flu A	NeuMoDx Flu B
69802	AH3Ν2	20.22	21.32	negative
69931	AH3Ν2	20.44	19.31	negative
70467	AH3Ν2	11.07	14.21	negative
70393	AH3Ν2	20.20	20.00	negative
70214	AH3Ν2	25.96	26.00	negative
70310	AH3Ν2	19.45	21.00	negative
70342	AH3Ν2	23.86	22.00	negative
70381	AH3Ν2	17.53	17.00	negative
70427	AH3Ν2	19.91	19.39	negative
89962	AH3Ν2	18.32	27.01	negative
89963	AH3Ν2	22.32	25.78	negative
89965	AH3Ν2	26.66	29.74	negative
89967	AH3Ν2	22.10	26.14	negative
89968	AH3Ν2	24.80	23.95	negative
89971	AH3Ν2	24.87	18.94	negative
89950	B	20.63	negative	28.68
89955	AH3Ν2	20.54	24.92	negative
89956	AH3Ν2	24.64	27.97	negative
89958	AH3Ν2	18.05	24.65	negative
89960	AH3Ν2	21.04	25.69	negative
89961	AH3Ν2	18.47	23.57	negative
89945	AH3Ν2	17.79	24.61	negative
89946	AH3Ν2	25.20	36.33	negative
89947	AH3Ν2	24.57	25.56	negative
89948	AH3Ν2	17.22	15.66	negative

**Table 2:** RSV RT-nested PCR positive samples tested on the NeuMoDx Flu A-B/RSV/SARS-CoV-2 Vantage Assay (*n* = 46).

Sample ID	Virus Type	NeuMoDx RSV	NeuMoDx SARS-CoV-2
83515	RSV-A OΝ1	22.54	negative
83521	RSV-A OΝ1	12.22	negative
83523	RSV-A OΝ1	17.01	negative
83524	RSV-A OΝ1	15.76	negative
83528	RSV-A OΝ1	17.17	negative
83529	RSV-A OΝ1	28.01	negative
83530	RSV-A OΝ1	21.42	negative
83531	RSV-A OΝ1	24.38	negative
83532	RSV-A OΝ1	16.36	negative
83533	RSV-A OΝ1	11.96	negative
83534	RSV-A OΝ1	23.73	negative
83535	RSV-A OΝ1	13.24	negative
83536	RSV-A OΝ1	16.36	negative
83537	RSV-A OΝ1	20.94	negative
83538	RSV-B BA	16.60	negative
83539	RSV-B BA	16.73	negative
83541	RSV-A OΝ1	26.91	negative
83542	RSV-A OΝ1	28.53	negative
83543	RSV-A OΝ1	20.62	negative
83545	RSV-A OΝ1	30.17	negative
83547	RSV-A OΝ1	18.24	17.21
83550	RSV-A OΝ1	24.30	negative
83552	RSV-A OΝ1	25.69	negative
83553	RSV-A OΝ1	29.42	negative
83556	RSV-A OΝ1	20.93	negative
83558	RSV-A OΝ1	28.64	negative
83562	RSV-A OΝ1	24.43	negative
83630	RSV-A OΝ1	12.56	negative
83564	RSV-A OΝ1	13.71	negative
83568	RSV-A OΝ1	31.50	negative
83570	RSV-B	negative	negative
83572	RSV-B BA	30.20	negative
83574	RSV-B BA	30.00	negative
83576	RSV-B BA	29.06	negative
83597	RSV-B BA	29.09	negative
83578	RSV-A OΝ1	32.11	negative
83580	RSV-B BA	23.16	negative
83582	RSV-A OΝ1	27.37	negative
83584	RSV-A OΝ1	28.00	negative
83586	RSV-B BA	30.03	negative
83588	RSV-A OΝ1	31.35	negative
83590	RSV-A OΝ1	26.39	negative
83592	RSV-A	negative	negative
83593	RSV-A OΝ1	28.97	negative
83594	RSV-A OΝ1	25.94	negative
83596	RSV-B	negative	negative

**Table 3:** Cycle threshold values of Abbott RealTime SARS-CoV-2 positive samples tested on the NeuMoDx Flu A-B/RSV/SARS-CoV-2 Vantage Assay (*n* = 44).

Sample ID	Abbott RealTime SARS-CoV-2	NeuMoDx SARS-CoV-2
49437	7.90	19.86
49519	8.77	21.13
45713	7.08	17.15
45714	4.51	15.54
45715	6.13	20.86
45329	8.80	20.93
45216	9.38	19.34
44819	6.67	15.08
44439	9.43	18.99
44061	5.26	15.68
44056	9.14	19.18
42996	5.20	16.73
42478	10.07	16.64
42415	10.27	20.64
42475	5.92	17.20
37996	5.56	15.67
37960	4.62	12.21
37636	8.77	16.21
37637	7.53	16.90
37416	9.16	21.64
37395	4.16	14.39
37400	5.85	16.27
37413	6.05	14.78
37360	2.91	12.45
37359	6.25	16.35
273620	18.77	31.04
273616	13.30	24.95
273557	19.51	29.72
273615	11.35	23.61
273245	18.27	28.55
273468	16.10	28.55
273457	15.56	26.75
273418	14.33	28.46
273237	12.89	21.48
273076	15.77	26.74
273072	15.00	27.62
272923	5.81	18.00
272565	15.45	27.02
271235	20.80	32.15
273555	9.98	19.41
271076	10.92	23.32
271027	8.83	19.32
271024	8.61	19.89
271023	7.81	21.03

## Data Availability

Not applicable.
